# Supplementary material for: Inflammatory factors and risk of lung adenocarcinoma: a Mendelian randomization study mediated by blood metabolites
Source: Front Endocrinol (Lausanne). 2024 Aug 27;15:1446863. doi: 10.3389/fendo.2024.1446863 (PMC11384989; doi:10.3389/fendo.2024.1446863)
Supplement: Supplementary file 1 [file DataSheet1.zip › supplementary files/S2/GCST90200146/sensitivity-analysis.pdf]

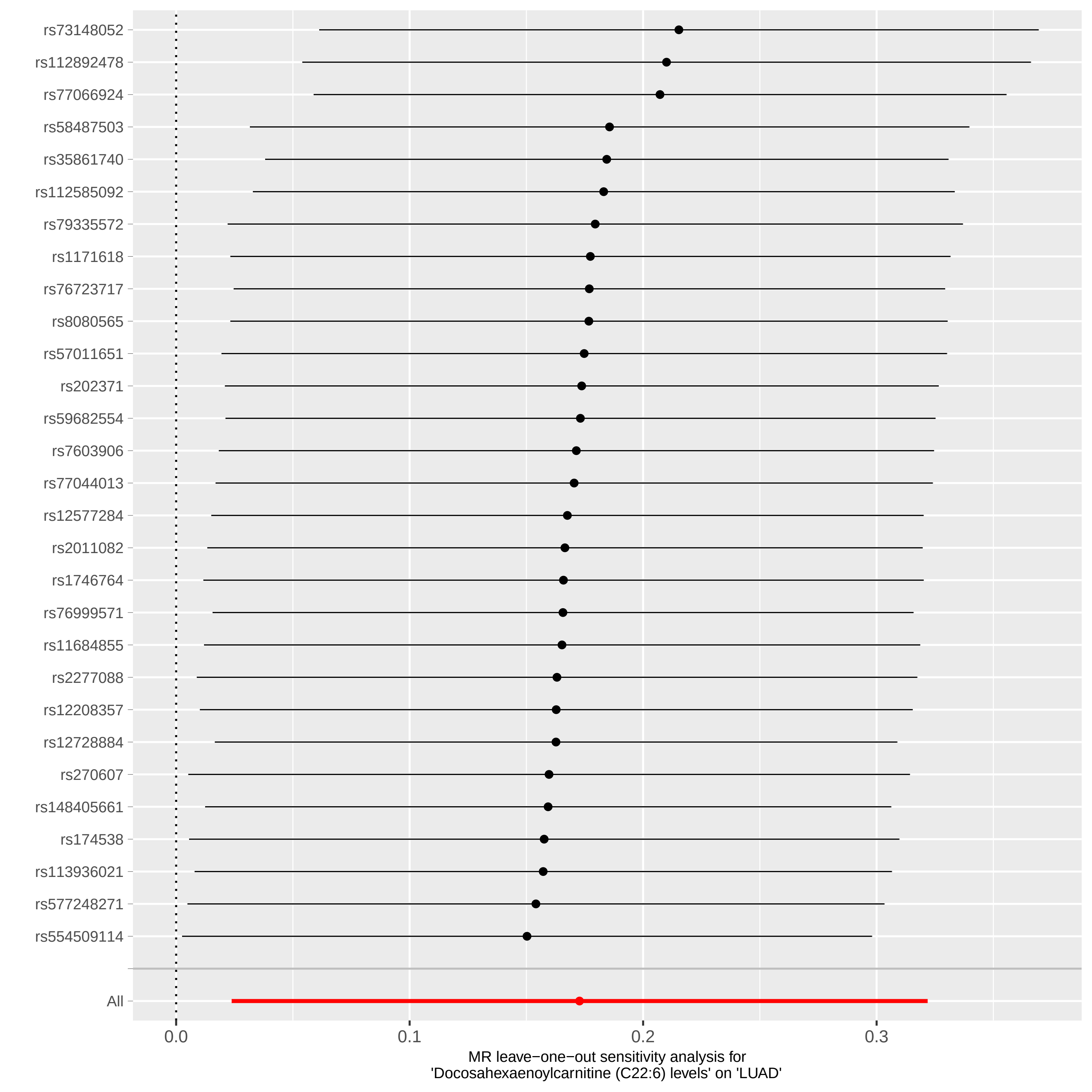

rs73148052
rs112892478
rs77066924
rs58487503
rs35861740
rs112585092
rs79335572
rs1171618
rs76723717
rs8080565
rs57011651
rs202371
rs59682554
rs7603906
rs77044013
rs12577284
rs2011082
rs1746764
rs76999571
rs11684855
rs2277088
rs12208357
rs12728884
rs270607
rs148405661
rs174538
rs113936021
rs577248271
rs554509114
All
0.0
0.1
0.2
0.3
MR leave−one−out sensitivity analysis for
'Docosahexaenoylcarnitine (C22:6) levels' on 'LUAD'
